# Primary patient-derived lung adenocarcinoma cell culture challenges the association of cancer stem cells with epithelial-to-mesenchymal transition

**DOI:** 10.1038/s41598-017-09929-0

**Published:** 2017-08-30

**Authors:** Verena Tiran, Joerg Lindenmann, Luka Brcic, Ellen Heitzer, Stefanie Stanzer, Nassim Ghaffari Tabrizi-Wizsy, Elvira Stacher, Herbert Stoeger, Helmut H. Popper, Marija Balic, Nadia Dandachi

**Affiliations:** 1 Division of Oncology, Department of Internal Medicine, Medical University of Graz, A-8036 Graz, Austria; 2 Division of Thoracic and Hyperbaric Surgery, Medical University of Graz, A-8036 Graz, Austria; 3 Institute of Pathology, Medical University of Graz, A-8036 Graz, Austria; 4 Institute of Human Genetics, Medical University of Graz, A-8010 Graz, Austria; 5 SFL Chicken CAM Lab, Institute of Pathophysiology and Immunology, Medical University of Graz, A-8010 Graz, Austria; 6 Ludwig Boltzmann Institute for Lung Vascular Research, A-8010 Graz, Austria; 7 Research Unit Circulating Tumor Cells and Cancer Stem Cells, Medical University of Graz, A-8036 Graz, Austria; 8 Research Unit Epigenetic and Genetic Cancer Biomarkers, Medical University of Graz, A-8036 Graz, Austria

## Abstract

The cancer stem cell (CSC) and epithelial-to-mesenchymal transition (EMT) models have been closely associated and used to describe both the formation of metastasis and therapy resistance. We established a primary lung cell culture from a patient in a clinically rare and unique situation of primary resistant disease. This culture consisted of two biologically profoundly distinct adenocarcinoma cell subpopulations, which differed phenotypically and genotypically. One subpopulation initiated and sustained in spheroid cell culture (LT22s) whereas the other subpopulation was only capable of growth and proliferation under adherent conditions (LT22a). In contrast to our expectations, LT22s were strongly associated with the epithelial phenotype, and expressed additionally CSC markers ALDH1 and CD133, whereas the LT22a was characterized as mesenchymal with lack of CSC markers. The LT22s cells also demonstrated an invasive behavior and mimicked gland formation. Finally, LT22s were more resistant to Cisplatin than LT22a cells. We demonstrate a primary lung adenocarcinoma cell culture derived from a patient with resistant disease, with epithelial aggressive subpopulation of cells associated with stem cell features and therapy resistance. Our findings challenge the current model associating CSC and disease resistance mainly to mesenchymal cells and may have important clinical implications.

## Introduction

Despite substantial recent progress in the treatment of lung cancer, in particular non-small cell lung cancer (NSCLC), the survival rate remains poor, with approximately 10–20% of patients surviving 5 years after diagnosis^1^. With the worldwide leading cause of cancer deaths in men (1.1 million males per year) and second leading cause in women (491,200 females per year) the necessity for further improvement in the treatment of lung cancer is clearly demonstrated^2^. These poor survival rates greatly strengthened the efforts to establish biomarkers for earlier diagnosis, prognosis and better treatment prediction. There are two major histological groups of lung cancer: 80–85% of tumors belong to the NSCLC, and 15–20% to the small cell lung cancer (SCLC). In the group of NSCLC the three major types are: adenocarcinomas (AC), squamous cell carcinomas (SCC) and large cell carcinomas. While smoking is strongly associated with an increased risk to develop SCLC or SCC, AC is not only the most common type of NSCLC, but also most commonly diagnosed in patients who have never smoked^3^.

Within primary tumors a small population of tumor cells has been identified possessing the capability of self-renewal and pluripotency^4^. As their characteristics are similar to embryonic stem cells they are termed cancer stem cells (CSC) or also tumor initiating cells. CSC are hierarchically organized and are capable of symmetric and asymmetric cell division^5^. The first evidence of CSC was observed in hematological malignancies^6^, and substantial literature on CSC also exists in various solid tumors^7–10^. Typically, CSC have been identified by expression of markers associated with stem cell properties. In NSCLC, CD133 and aldehyde dehydrogenase 1 (ALDH1) have been described as candidate markers for enrichment of CSC. Prognostic impact of cells expressing these markers has already been demonstrated^11^. Although CD133^+^ cells found in tumor samples from lung cancer patients constitute a minor population, their tumorigenic potential has been proven in an animal model^12^. While the CD133 function is still unclear^13^, a high occurrence of ALDH1 can be related to therapy resistance, as it oxidizes a large number of aldehydes. ALDH1 is also associated with processes of proliferation and survival^14^. Both markers are associated with aggressive behavior of the tumor, poor prognosis and cancer recurrences^15, 16^.

A further trait of CSC is their capability to metastasize, as we and others have shown^17, 18^. Epithelial-to-mesenchymal transition (EMT) has been closely associated with CSC as one of the mechanisms facilitating the formation of metastasis^19^. EMT is a well-known process occurring during the embryonic development for tissue morphogenesis and in cancer, EMT has been correlated to CSC plasticity^20^. During the metastasis formation tumor cells are losing their cell-cell contact supposedly accompanied with a downregulation of E-Cadherin (ECad) and epithelial markers such as the epithelial cell adhesion molecule (EpCam), cytokeratin (CK), and an upregulation of Vimentin^21^. This metamorphosis allows the cells to escape from the epithelial compartment and to proceed to distant sites. Unfortunately, there is only limited clinical data and experimental models providing convincing evidence. Therefore, the effect of EMT for metastasis formation and association with stemness is still a matter of debate^22^. A recent study in mouse models with different tumor entities showed that metastases are derived from non-EMT tumor cells^23, 24^. Overall, most data supporting either the EMT theory or the opposite were derived from cell lines and mouse experiments, which are not sufficiently able to capture this transient mechanism. Clinical data from patients proving or disproving that EMT is necessary for metastasis formation is scarce.

Here, we show our established primary cell culture from a patient diagnosed with adenocarcinoma of the lung resistant to conventional treatment, which we believe to challenge the current model associating CSC with EMT and therapy resistance.

## Results

### Isolation and propagation of primary lung cancer adenocarcinoma cells

**Figure 1:**
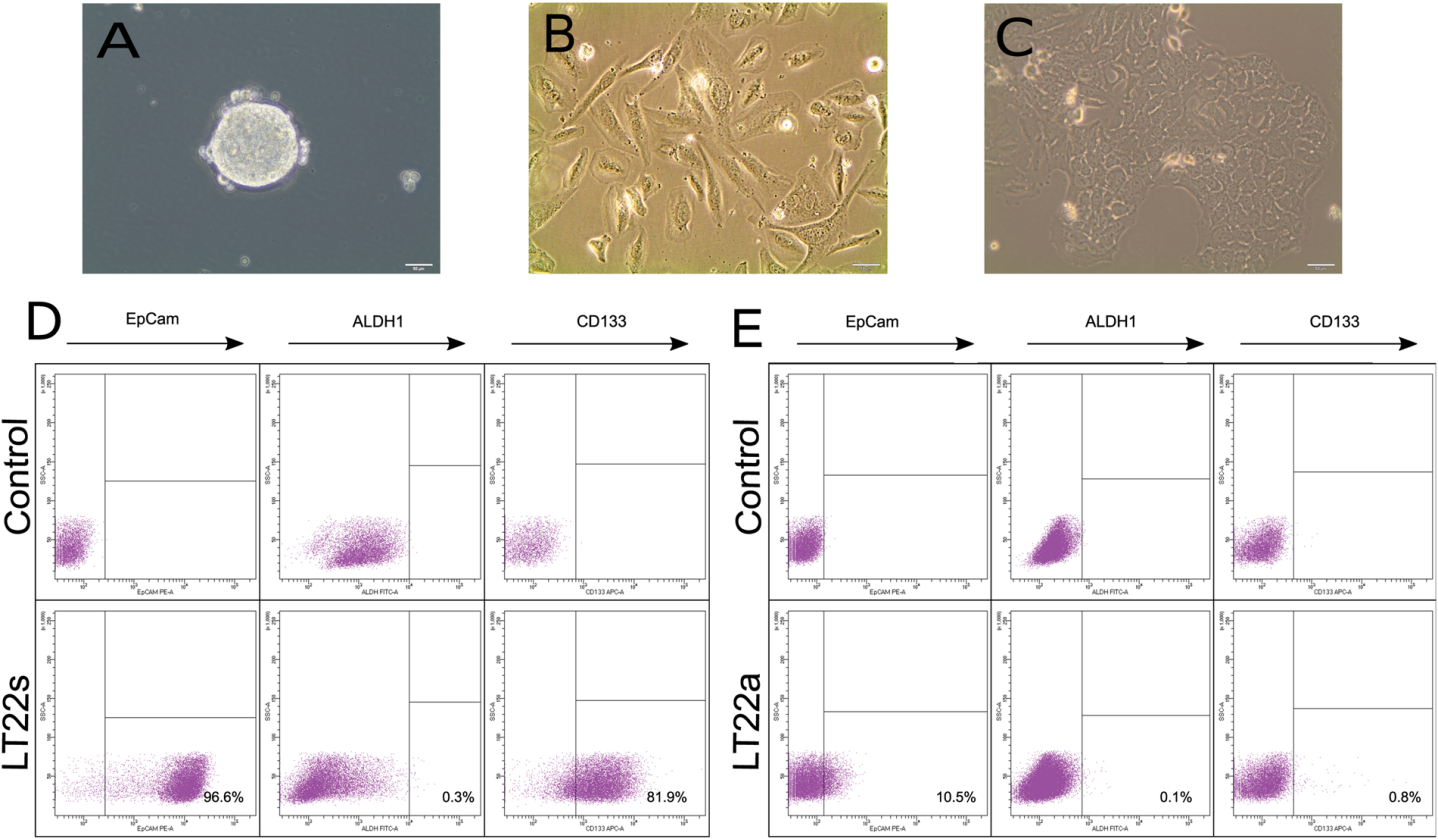
Isolation and identification of two distinct cell subpopulations of a patient-derived primary lung adenocarcinoma with distinct phenotypes. Phase-contrast images of LT22s cells cultured under spheroid culture conditions (**A**), LT22a cells cultured under adherent culture conditions (**B**) and (**C**) LT22s cells transferred into adherent culture after 18 spheroid passages. Flow cytometry based identification of CSC markers in LT22 cells showing (**D**) LT22s and (**E**) LT22a cells expressing EpCam, ALDH1 and CD133. Controls were stained with isotypes or treated with DEAB.

After isolating a single cell suspension of the primary lung adenocarcinoma tumor, cells were cultured in non-adherent culture conditions, referred to as LT22s, and 10 days were needed for the formation of spheres, as shown in Fig. 1A. During the first passages, the cell culture consisted of a heterogeneous mixture of spheres and single cells. Therefore, we decided to initiate an adherent culture supplemented with FBS after the second passage, hereinafter referred to as LT22a. This adherent cell culture was fast-growing and contained a large proportion of spindle shaped cells which were not able to reach 100% confluency. These cells showed various shapes and sizes and displayed loose contact with neighboring cells (Fig. 1B). In later passages (>passage 19) we evaluated the capacity of LT22a to grow spheres, but without success. However, the generation of colonies in soft agar matrices was possible, suggesting the necessity of an extra cellular matrix to grow spheroids. We also tried to bring later passages (>passage 15) of LT22s into adherent culture. These cells appeared as cobblestone-like cells with close contact to other cells, as represented in Fig. 1C. Their proliferation was much slower in comparison to the LT22a spindle shaped cells (data not shown).

### Enrichment of cancer stem cell markers

The presence of putative CSCs was investigated with FACS using CD133 and ALDH1. LT22s showed a continuous increase of the putative cancer stem cell marker CD133 with serial passages, starting from 14% and resulting in more than 80% after passage 19. Additionally, EpCam reached a constant expression of more than 90% (starting from 58%). In contrast, ALDH1 expression was not very prominent in LT22s cells at the beginning (mean 8.4% ± 1.4% SD) and even decreased with serial passages (mean 0.4% ± 0.3% SD) (Fig. 1D). Due to this rather low ALDH1 expression, no sorting experiments were performed with this subpopulation. In the LT22a culture, the expression of EpCam was 50% in the beginning and decreased over time until it reached a constant expression with an average of 9.4% (±2.7% SD). In the adherent cell culture, the ALDH1 and CD133 expression was very low with less than 1–2% (Fig. 1E). Based on these numbers, the sorting experiments were performed with CD133 in the LT22s culture and with EpCam in the LT22a culture.

### Characterization of LT22 primary tumor and cells of both culture conditions

For genotypic and phenotypic characterization of the LT22s and LT22a cultures, qRT-PCR and immunostaining was performed, respectively. The expression of selected markers in the cell culture was compared to the respective formalin-fixed paraffin-embedded (FFPE) primary tumor. For the analyses of the primary tumor, only IHC was used.

**Figure 2:**
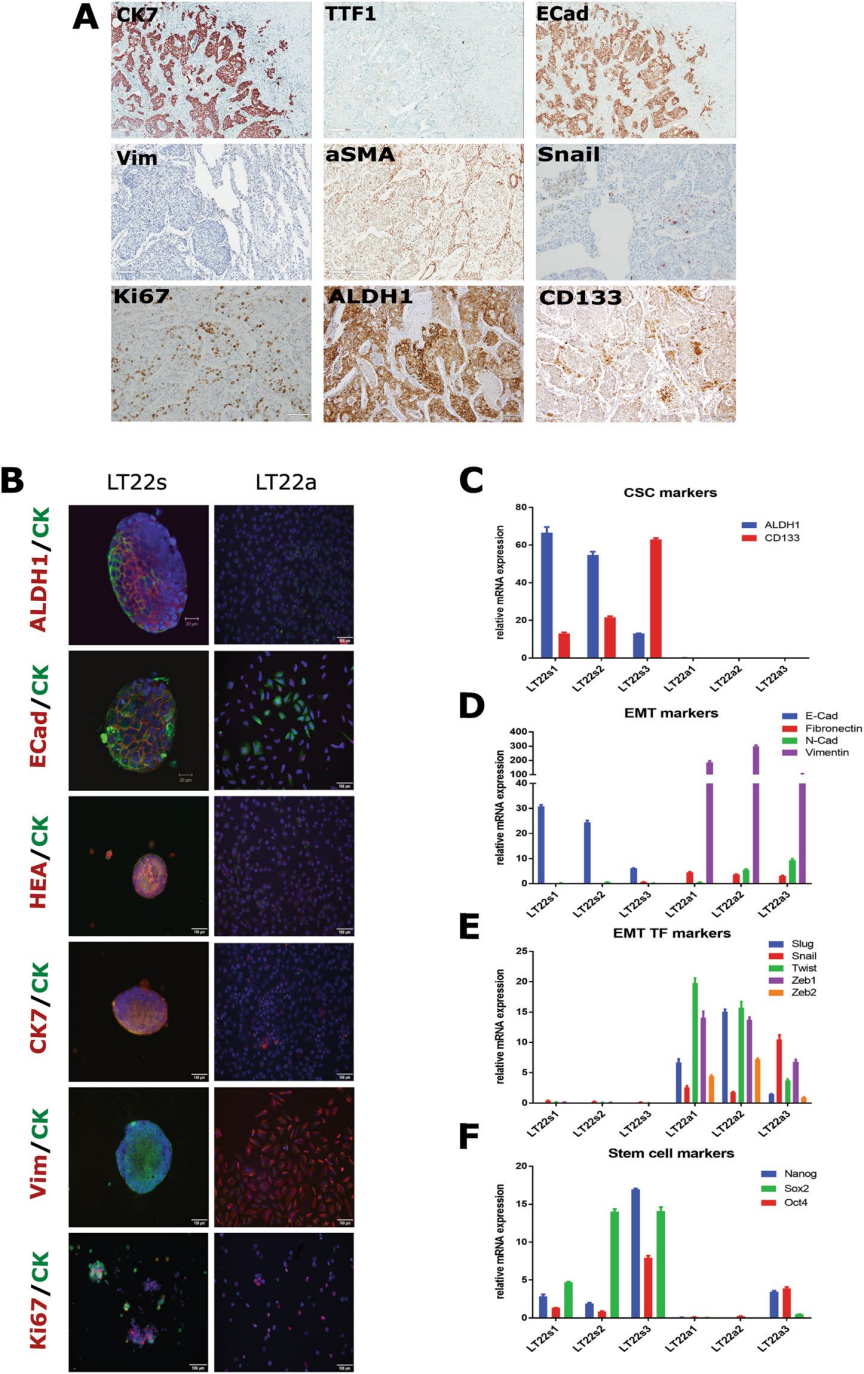
Phenotypic characterization of the primary tumor and LT22s and LT22a cells using immunostaining techniques and qRT-PCR. (**A**) IHC staining images of diagnostic, EMT and CSC markers on paraffin sections of the primary tumor. Immunofluorescent images of EMT and CSC markers of (**B**) LT22s (left) and LT22a (right) cells. Red channel ALDH1, ECad, HEA, CK7, Vimentin and Ki67; blue channel DAPI, green channel panCK and all channels merged. Relative gene expression of LT22s and LT22a cells. Data are obtained from three different passages (LT22s 1–3 and LT22a 1–3) and represented as mean ± SEM. Bar graphs show (**C**) CSC markers, (**D**) EMT associated markers and (**E**) EMT transcription factors, and (**F**) stem cell associated markers.

IHC staining of the primary tumor revealed following characteristics summarized in Fig. 2A: the tumor was diagnosed as adenocarcinoma due to a positive staining for CK7, but negative for thyroid transcription factor 1 (TTF1). Further characterization of EMT markers revealed that E-Cadherin staining was positive, whereas Vimentin and alpha-smooth muscle actin (aSMA) were negative within the tumor. There were few single cells, which stained positive for the EMT transcription factor Snail. With 60% of cells positive for Ki67, the tumor was highly proliferating. Furthermore, both CSC markers ALDH1 (40%) and CD133 (20%) were expressed in the primary tumor.

Corresponding marker analysis was performed in LT22s and LT22a summarized in Fig. 2B. In LT22s 80% of cells expressed the cancer stem cell marker ALDH1 and 10% of cells CD133. Therefore, the CSC fraction in LT22s cells was enriched with ALDH1-positive cells compared to the primary tumor. In contrast, neither ALDH1 nor CD133 expression could be detected in LT22a cells. Additional staining was performed to further characterize LT22s and LT22a. First, we analyzed the expression of representative epithelial and mesenchymal markers. Epithelial markers, including ECad, EpCam, panCK, and in particular CK7 were clearly positive in LT22s, whereas in LT22a, a weak expression of epithelial markers could be detected, with only few cells positive for CK7 (10%). The mesenchymal marker Vimentin was expressed in 90% of the LT22a cells, indicating a mesenchymal phenotype. In contrast, neither the primary tumor sample nor LT22s showed expression of Vimentin. However, the highly proliferative rate seen in the primary tumor was sustained under both culture conditions LT22s and LT22a with up to 60% positive for Ki67.

Results from qRT-PCR supported the results of the immunostainings. In LT22s cells, CD133 expression increased with serial passages as shown in Fig. 2C. Although ALDH1 mRNA expression decreased over passages, it was still prominent at the protein level, as shown by immunofluorescence staining. Concordantly, ECad mRNA levels were also upregulated. We did not detect any expression of mesenchymal markers or EMT transcription factors in LT22s, which is in line with an epithelial phenotype.

In concordance with the immunostaining results, no mRNA expression of the cancer stem cell markers ALDH1 and CD133 could be detected in LT22a cells. While the expression of ECad was not detectable, mesenchymal markers such as Vimentin, NCadherin (NCad) and Fibronectin were increased (Fig. 2D). EMT transcription factors, including Slug, Snail, Twist, Zeb1 and Zeb2, were upregulated at the mRNA level (Fig. 2E). These findings once more underline the mesenchymal phenotype of the LT22a cells, which could not be found within the primary tumor.

Finally, stem cell associated markers Oct4, Sox2 and Nanog were analyzed by qRT-PCR (Fig. 2F). These markers were upregulated in LT22s, with subsequent increase over passages. In contrast, there was little or no expression of these stem cell markers in LT22a.

Taken together, these results confirm the epithelial and stem-cell associated phenotype of LT22s cells and the mesenchymal phenotype of LT22a cells at both the mRNA and at the protein level. These results are also consistent with the morphologic characteristics of the two cell populations.

### CNV patterns of primary tumor, LT22s and LT22a cells

**Figure 3:**
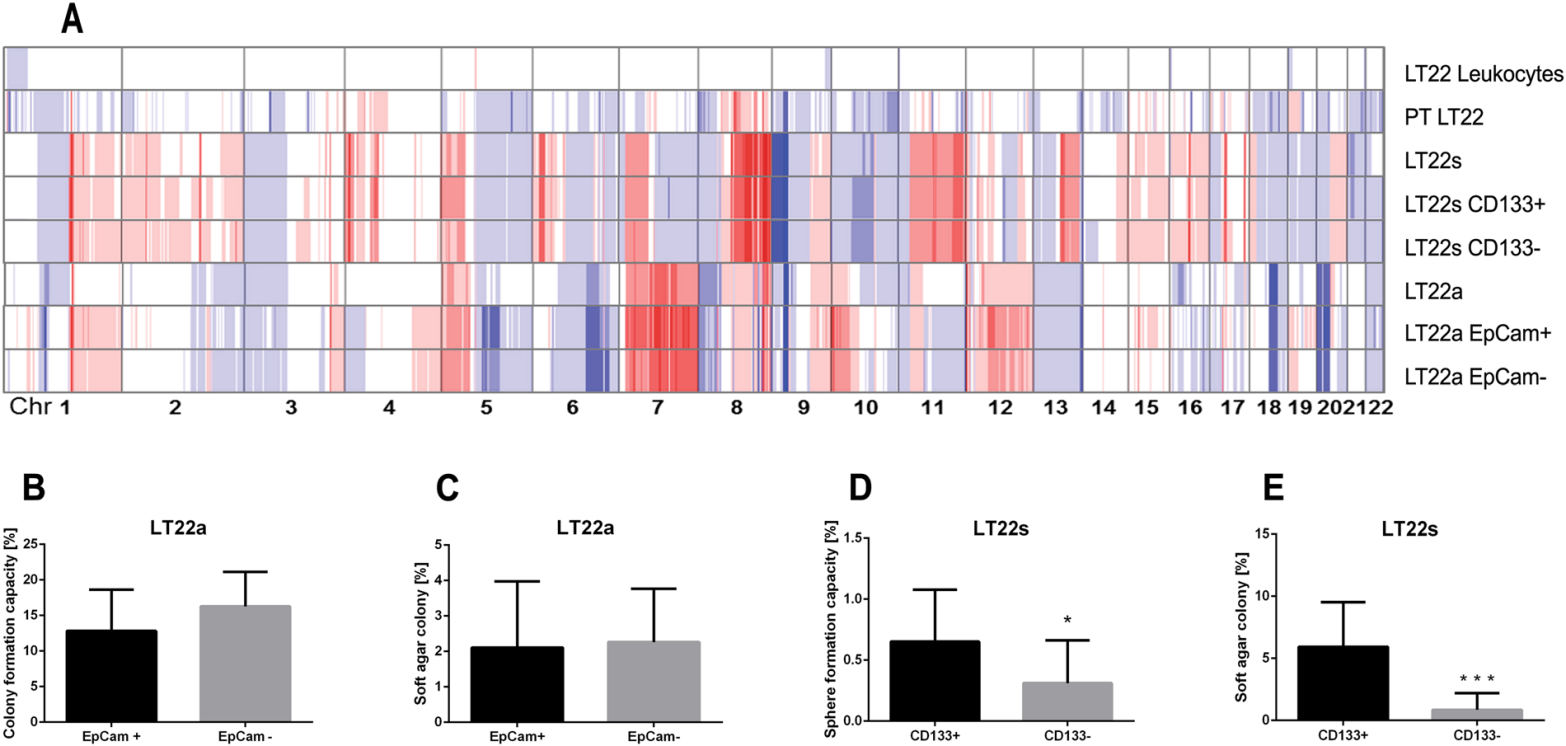
Genetic profiles and *in vitro* assays of LT22s and LT22a cells. (**A**) Genetic heat map of copy number profiles from low-coverage whole genome sequencing of primary tumor (PT) and subpopulations. Blue color indicates loss of chromosomal material, red color indicates gain of chromosomal material. Balanced regions are shown in white. EpCam sorted LT22a cells were evaluated for (**B**) colony formation capacity and (**C**) colony formation capacity in soft agar. CD133 sorted LT22s cells were evaluated for (**D**) sphere formation capacity and (**E**) colony formation capacity in soft agar. Experiments were done with three biological replicates. (*P < 0.05 and ***P ≤ 0.0001 Students t-test).

In order to assess whether genetic changes underlie the different phenotypes of LT22a and LT22s, we employed low-coverage whole genome sequencing to establish genome-wide somatic copy number alterations (SCNA), which is pictured in Fig. 3A. Additionally, the leukocyte fraction and the primary tumor were analyzed. As expected the leukocyte fraction showed a balanced copy number profile. In contrast, several recurrent SCNAs were detected in the primary tumor and both LT22s and LT22a, such as the amplifications on chromosomes 1p, 5p, and 7p, and deletions on 3p, 8p, and 17p that are frequently observed in lung adenocarcinoma^25^. These multiple aberrations suggest dysregulations of biological functions and confirm the tumorigenic potential. It is of note that aberrant regions were less prominent in the primary tumor than in the cultured cells. Most likely these additional changes result from potential somatic mosaicisms in the primary tumor that were cloned during the establishment of the cell cultures. Interestingly, the SCNA patterns of LT22s compared to LT22a were also dissimilar, indicating that substantial chromosomal alterations contribute to the different phenotypes. The most prevalent differences included 2q, 4p, 6p, 7q, 8q, 9p, 10p, 11, 13q and 20q. However, when we analyzed the two cell populations after cell sorting based on CD133 expression for LT22s and EpCam expression for LT22a, we did not observe a difference between the two cell fractions, indicating that the expression of these markers is driven by other mechanisms than SCNAs, such as point mutations or regulatory mechanisms on the transcriptional level. In order to prove that LT22a and LT22s indeed originate from the same patient, we performed STR analysis and comparisons of mitochondrial haplotypes. Both LT22a and LT22s showed the same STR profiles and mitochondrial SNP patterns than the primary tumor and constitutive DNA of the patient retrieved from leukocytes confirming a common origin (Supplementary Table S1).

### *In vitro* assays

As mentioned before, LT22a cells did not have the capability to form spheres under non-adherent conditions. This observation is in line with the fact that these cells did not form tight contacts in adherent culture and the lack of cell adhesion marker ECad. Together, this data supports the mesenchymal phenotype of LT22a cells. On the other hand, when LT22s was transferred to adherent cell culture, a large number of cells were needed in order to enable attachment and growth of cells.

Next, we investigated in LT22a whether the expression of EpCam had an influence on their ability to form colonies in soft agar conditions. However, both EpCam^+^ and EpCam^−^ subpopulations formed colonies to a similar extent (Fig. 3B and C). The LT22s cells were sorted for the putative cancer stem cell marker CD133. Both the sphere formation assay and the soft agar assay showed a higher sphere formation capacity of cells expressing CD133^+^ (Fig. 3D and E) compared to CD133- cells. In soft agar assay this difference was highly significant (*P* = *0*.*0001*). These results indicate that CD133^+^ cells have a higher tumorigenic potential than CD133- cells.

### Cell formation in an ex ovo system

**Figure 4:**
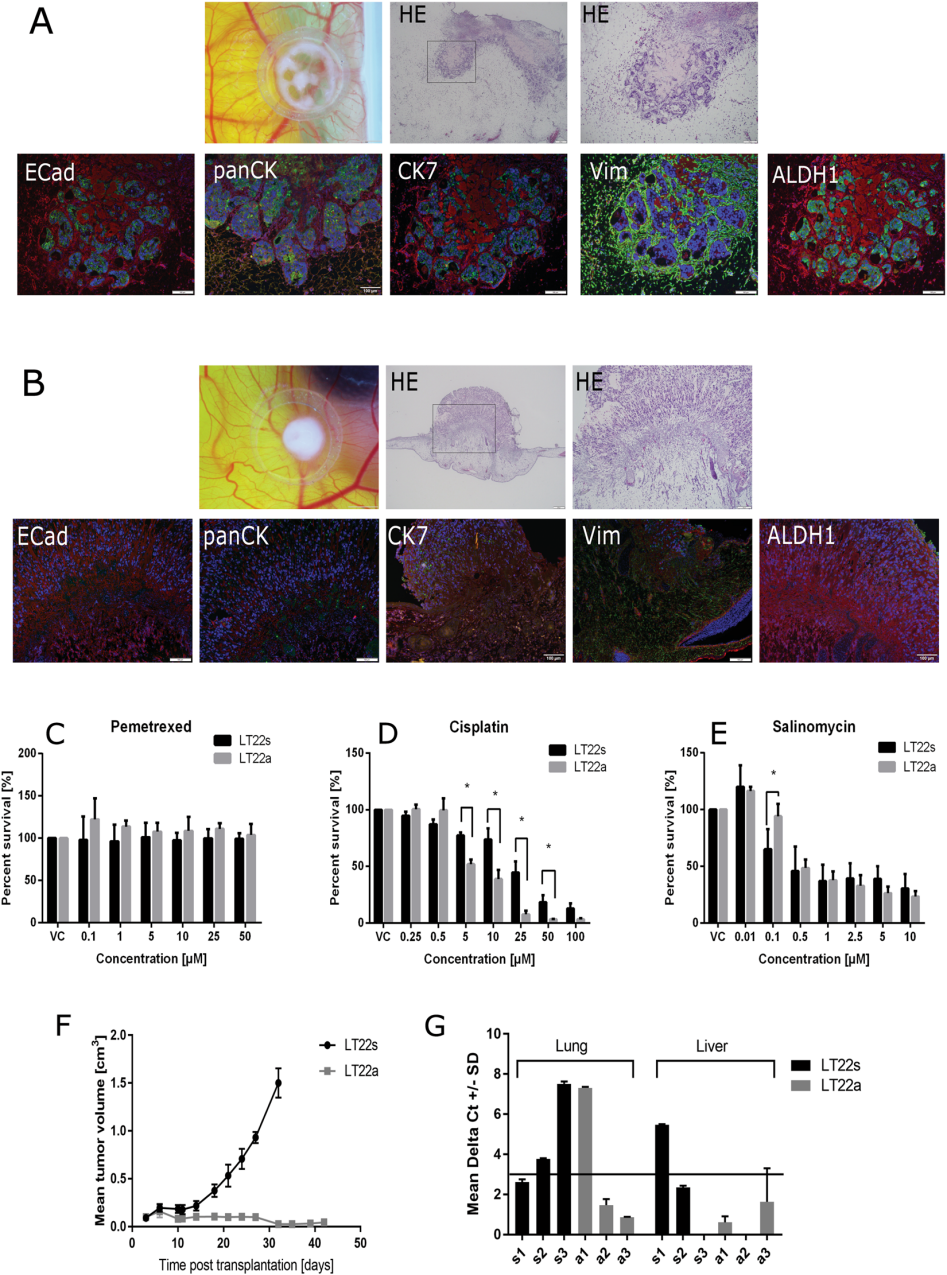
*In vivo* experiments with LT22a and LT22s cells. *In vivo* CAM assay with (**A**) LT22s and (**B**) LT22a cells. Upper panel: Macroscopic view of on-plant. Measuring bar 2 mm. Hematoxylin eosin (HE) staining of on-plant. Measuring bar 200 µm and 100 µm. Lower panel: Immunofluorescent staining with EMT and CSC markers. Measuring bar 100 µm. In drug treatment assays LT22s and LT22a cells were incubated with varying concentrations of Pemetrexed (**C**), Cisplatin (**D**) and Salinomycin (**E**). Cell viability was measured with Prestoblue assay and percent viability was normalized to vehicle controls (VC). Data are represented as mean +/− SEM. Experiments were done in five technical replicates and three independent biological replicates. (*P < 0.05 ANOVA with Sidak multiple comparison) Female NOD/SCID mice were transplanted at day 0 with 10^7^ cells of patient derived cells from adenocarcinoma. (**F**) Shown is the mean tumor volume +/− SD for three mice/cell line over time. (**G**) Micro metastasis in lung and liver tissues were evaluated with PCR. This bar graph summarizes ΔCt value +/− SD for liver and lung tissue (n = 2) as indicator for micro metastases of LT22s and LT22a cells. The horizontal line indicates the cut-off value (ΔCt = 3.0) for metastasis, with values >3.0 indicating metastasis.

The tumorigenicity of the LT22a and LT22s cells was also tested in an *ex ovo* chorioallantoic membrane (CAM) assay model, a well-established *in vivo* tumor system^26, 27^. One advantage of this model is the independence of the culture conditions, because the cells grow directly in the CAM microenvironment. The LT22s and LT22a cells were applied with 1*10^6^ single cells per on-plant onto the CAM of the chicken embryo to form tumors. The tumor formation differed at both the macroscopic and microscopic level. After 72 hours on CAM the LT22s cells formed a compact round-shaped structure whereas the LT22a showed a centrum with radial arranged branches at macroscopic level (Fig. 4A and B). Hematoxylin eosin (HE) staining showed an invasion through the membrane in both cases, indicating an invasive potential of both cell types. For the LT22a cells, the tumor consisted of spindle-shaped cells, which infiltrated the membrane. A structural formation could not be observed with the cells randomly arranged around the chicken membrane. The LT22s cells also invaded the membrane but formed glandular structures (Fig. 4A) recapitulating the primary lung adenocarcinoma.

The immunofluorescent staining revealed that LT22s cell clusters had a positive staining for ECad, ALDH1, panCK and CK7 consistent with the parental cell line. Vimentin was negative in tumor cells, but stained the mesenchymal cells of the chicken egg (Fig. 4A). In contrast, LT22a cells stained negative for ECad, panCK and ALDH. While only few cells stained positive for CK7, Vimentin was positive in all cells (Fig. 4B). Taken together, these results correspond to the immunostaining results of the primary parental cells *in vitro*. No phenotypic changes could be observed under the influence of the microenvironment of the chicken egg.

### Drug treatment viability assay

Since CSC are held responsible for therapy resistance^28^, we measured the cytotoxicity of LT22a and LT22s cells upon exposure to various drugs. First, we tested influence of Salinomycin, which has been shown to be specifically toxic to stem-like cancer cells, on the viability of the cells^29^. Additionally, two common anticancer drugs (Pemetrexed and Cisplatin) for lung adenocarcinoma were used for viability assays. In both LT22a and LT22s, Pemetrexed did not reduce viability at any concentration tested (Fig. 4C). However, Cisplatin decreased viability with increasing drug concentrations with a higher efficacy on LT22a cells (IC50 5.97 µM) compared to LT22a cells (IC50 18.91 µM) (Fig. 4D) Salinomycin also decreased viability in both LT22a and LT22s with increasing drug concentration (Fig. 4E). This effect was slightly stronger in LT22s, which is also noticeable in IC 50 values; 0.68 for LT22s compared to LT22a cells with an IC50 value of 0.80.

### *In vivo* mouse experiments

Finally, to assess the *in vivo* tumorigenic potential of LT22s and LT22a cells, we performed subcutaneous injection of cells in severe combined immunodeficiency (SCID) mice. Both cell lines started to form tumors, but LT22s cells were significantly more tumorigenic *in vivo* compared to LT22a cells (Fig. 4F). LT22s cells formed tumors rapidly, while LT22a cells grew much slower and tumor growth remained steady at 0.1 cm^3^. After histological examination of the tumor tissues, we observed morphologic differences of the LT22s and LT22a tumors, similar to those seen with CAM assays. LT22s cells formed tumors with morphological features closely resembling the original tumor (Supplementary Fig. S1). In addition, immunohistochemistry staining revealed that both LT22s and LT22a cells maintained antigen expression as described *in vitro* (Supplementary Fig. S1).

In order to assess the metastatic potential of both cell lines, human specific PCR was used to determine the presence of human cells in liver and lung (micrometastases). PCR revealed the presence of human cells in liver and in lungs in both groups of mice, but to a different degree. In two (A1 and A2) out of the three mice of group A transplanted with LT22s cells we detected micrometastases in liver and lung, while in the mouse A3 only micrometastases in the lung were detected, but to a relatively strong extent (Fig. 4G). In both mice groups, metastasis into lung were more pronounced than into liver. Interestingly, in LT22a derived tumor bearing mice we also detected micrometastases despite their low tumorigenic potential, as described above. Overall, LT22s cells revealed a higher metastatic potential compared to LT22a cells.

## Discussion

With the established primary cell culture, our study challenges the current model associating CSC with EMT and disease resistance mainly to mesenchymal cells. Our cell culture was established from a patient diagnosed with adenocarcinoma of the lung, who was resistant to standard neoadjuvant treatment including Cisplatin, Docetaxel, Vinorelbine, and radiotherapy that revealed two distinct subpopulations of cells with biologically distinct features.

Contradicting our expectations, epithelial subpopulation of cells associated with CSC features, higher expression of stem cell marker genes, aggressive phenotype, enhanced invasion, and therapy resistance. Less aggressive cells, only capable of growth in adherent conditions, lacking stem cell and epithelial markers, and more responsive to treatment, were largely of mesenchymal origin.

Our data challenge the CSC model associated with the EMT phenotype^30, 31^. The presented cell culture model was generated from a patient in a rare clinical situation since the surgery was eventually performed with palliative intention, while disease progressed on conventional neoadjuvant treatment, including both chemotherapy and radiotherapy. Surprisingly, epithelial cells turned out to be more resistant than the mesenchymal subpopulation, implicating that future research of resistance should also focus on epithelial cells.

Over serial passages we observed the enrichment of cells with stem cell markers including ALDH1 and CD133 (LT22s cells), respectively. However, the expression of both proteins varied between the used methods, i.e. FACS and immunostaining. Although the anti-CD133 antibody was used from the same clone, it was differently labeled. Moreover, this antibody was specifically designed for FACS analysis, which might be another explanation for the detected discrepancy. ALDH1 staining for FACS analysis results from an enzymatic reaction. Various reasons such as inactivity of ALDH1 or the presence of inhibitors can influence the fluorescent reaction. Here, we observed even greater discrepancy between the immunostaining and FACS. Thus, we confirmed the enrichment of ALDH positive cells at mRNA level which correlated with the results of immunofluorescence, as shown in Fig. 2B and C.

In sharp contrast to the expected association between CSC and EMT, LT22s cells enriched for CSC were positive for epithelial markers including EpCam, CK and ECad, but lacked mesenchymal traits. The epithelial CSC phenotype was also consistent during the invasion process of the CAM assay. We could demonstrate that LT22s cells invaded the membrane and formed glandular structures with an inner lumen, which was comparable to the primary tumor cell formation and were also capable to form peripheral metastasis. Thereby, the expression of epithelial and CSC markers persisted. Therefore, it seemed that these cells were able to invade without undergoing EMT.

In addition to the epithelial CSC-enriched spheroid culture (LT22s), we also isolated mesenchymal-like cells of the same tumor, with a high Vimentin expression and a prominent mesenchymal phenotype. These LT22a cells did not express any CSC markers, but revealed a spindle-shaped morphology together with strong expression of mesenchymal markers at protein and mRNA level. We also found that LT22a cells were not able to proliferate and form spheres under spheroid culture conditions. The origin of LT22a cells was rather unclear, because in the primary tumor a Vimentin positive cell subpopulation could not be detected. However, in the primary tumor we found single cells positive for the EMT transcription factor Snail. We therefore hypothesize that these Snail-positive cells may be the origin of the LT22a culture. Under adherent conditions these cells might have gained a considerable growth advantage in the absence of LT22s cells. Furthermore, Snail has been shown to be responsible for downregulation of ECad^32^. The absence of epithelial cell adhesion markers including ECad could be one reason for the inability to form spheres. This is in line with a recent study, showing that sphere formation in breast cancer cell lines depends on expression of ECad^33^.

Due to these remarkable divergences of the two cell subpopulations, we evaluated their genomic relations. STR analysis and mtDNA analysis confirmed that both subpopulations originated from the same patient. Furthermore, genome-wide copy number profiling indicated that both subpopulations were highly degenerated, consistent with a malignant phenotype of these cells. However, SCNAs differed between epithelial and mesenchymal cells, further confirming the presence of two independent subpopulations of cells in the primary tumor.

Unfortunately, material from metastasis of this patient was not available, thus it remains unclear whether LT22s or LT22a cells were responsible for the formation of metastasis. We could, however, observe in mouse transplantation a higher potential of LT22s cells to form metastasis. Furthermore, a consistent epithelial phenotype of LT22s cells could be observed during invasion into the amnion cavity of the chick embryo, which further questions the necessity of EMT for metastasis. Recently published papers share our observations^34^. Fischer *et al*. generated a triple transgenic mouse model, where the EMT initiator fibroblast specific protein 1 (FSP-1) served as a switch for a Cre fluorescent alternation, which was not reversible. Cells with epithelial phenotype emitted a red fluorescence, but when EMT occurred, cells emitted a green fluorescence. Surprisingly, cells that traveled to the lung showed a red fluorescence staining and therefore kept their epithelial phenotype during the formation of metastasis^23^. Similarly, Zheng *et al*. generated a mouse model of pancreatic cancer to study the influence of EMT. They found that loss of either Snail or Twist suppressed EMT in the primary tumor, but did not reduce invasion and metastasis^24^. These two seminal papers seriously challenge the role of EMT as a key element involved in cancer metastasis. Together with our observation, these studies question the association of EMT and CSC and formation of metastasis. Nevertheless, it is important to mention that EMT is a dynamic and transient process and therefore, experiments to capture this complicated process are challenging. Interestingly, LT22a cells also formed metastasis to a minor extant, although *in vivo* tumor growth was limited.

Both previously mentioned papers suggest, on the other hand, that EMT contributed to drug resistance. This suggestion could not be confirmed with our experiments. In fact, LT22a cells showed a significantly better response to Cisplatin than LT22s cells. It is of note that the cell culture was established at a stage of disease progression under primary treatment with potentially curative intention. The surgery was performed as a salvage treatment due to the young age of our patient. Unfortunately, soon after the surgery our patient died. Therefore, this cell culture is representative for disease resistance resulting from a rare and unique clinical situation. Patients who progress under the neoadjuvant or definitive radiochemotherapy represent the minority, and those who undergo surgery are individual cases. Thus, the limitation of our study (that this observation is obtained from only one case) is at the same time its strength, since it is representative of progressive disease, where conventional therapies were ineffective.

Overall, we show for the first time the independence of CSC markers and EMT features in primary cells derived from a lung cancer patient resistant to conventional treatment. We believe that our data provide important clinical evidence challenging the current model of EMT in cancer progression, resistance and metastasis. Our data suggest that epithelial tumor cells play a crucial role in progression, therapy resistance, and metastasis, and have to remain in focus of further studies.

## Materials and Methods

Detailed methods can be found in the supplementary materials.

### Patient

A 43-year old male patient was diagnosed with a poorly differentiated adenocarcinoma of the lung in September 2014. No genomic aberrations for lung cancer-specific genes EML4-ALK and EGFR were detected. The patient received two cycles of neoadjuvant chemotherapy with Cisplatin (75 mg/m^2^) and Docetaxel (75 mg/m^2^) and during concomitant radiochemotherapy with Cisplatin (75 mg/m^2^) on day 1 and Navelbine (12.5 mg/m^2^) on days 1, 7 and 15 of the cycle. At the time of surgery lumbar bone and liver metastasis were suspected and the primary tumor progressed on treatment. The surgery was performed as a palliative treatment. Soon after the surgery, the patient had a further disease progression and did not receive any further systemic tumor specific treatment. The overall survival from the diagnosis to death was 6 months.

The present study was approved by the ethics committee of the Medical University of Graz (vote #26-073 ex 13/14). Informed consent for the primary cell culture was obtained from the patient. All experiments were carried out in consideration of the guidelines for good scientific practice as officially required from the Medical University of Graz.

### Primary cell isolation and culturing

Tumor cell isolation was performed as previously described with the addition of dissociation steps using a gentleMACS automated dissociator (Miltenyi Biotec, Bergisch Gladbach, Germany) and some modifications^35^. Isolated cells were cultured in ultra-low attachment flasks containing DMEM/F12 media (Gibco by life technologies, Carlsberg, CA, USA) supplemented with human recombinant EGF (20 ng/mL), bFGF (20 ng/mL; both Peprotech, Vienna, Austria), B27 (Gibco by life technologies, Carlsberg, CA, USA) and 4 µg/mL Heparin (Gilvasan, Vienna, Austria). The media was changed twice a week. At the appearance of brown spots in the center of the spheroids, they were dissociated with TrypLE (Gibco by life technologies). After two spheroid passages, a proportion of the cells was further cultured in adherent conditions, with DMEM/F12 media and 10% fetal bovine serum superior (FBS) (Biochrom, Berlin, Germany) in standard flasks (Costar, Sigma Aldrich, St. Louis, MO, USA).

### Flow cytometry staining and sorting

Staining of the cells for flow cytometry analysis was performed following two different protocols as adapted from our previous publication^36^. For the first staining, single cells were blocked with 0.5% bovine serum albumin/PBS, for 10 min at room temperature (RT). 1*10^6^ cells were incubated with aliquots of antibodies (5 µL anti-EpCam Phycoerythrin (PE) and 10 µL anti-CD133 Allophycocyanin (APC)) for 30 minutes at 4 °C. Another approach was to measure the ALDH1 activity with Aldefluor assay kit (Stemcell Technologies, Cologne, Germany), performed according to the manufacturerʼs instructions. All monoclonal antibodies were pretitered to determine their optimal dilutions before use. Flow cytometry sorting was performed on the fluorescence activating cell sorter (FACS) Aria (BD Bioscience, Schwechat, Austria). Cells without staining, single stained cells, and isotype controls (BD Bioscience) were used as controls in the experiments. Side scatter and forward scatter profiles were used to eliminate cell doublets and apoptotic cells were excluded by using 7-aminoactinomycin D (AAD; BD Bioscience). Data analysis was performed using the Diva software 7.0 (BD Bioscience). Sorted cells were seeded into ultra-low attachment flasks for evaluation of sphere formation capacity. Additionally, DNA and RNA were extracted from sorted fractions for sequencing and gene expression analyses.

### Multicolor Immunofluorescence

The expression of selected cancer related proteins was analyzed with multicolor immunofluorescence staining. Cytospins from single cells or compact spheres, chamber slides with adherently grown cells (Lab-Tek, Sigma Aldrich, Vienna, Austria) and adhesion slides (Marienfeld, Lauda-Königshofen, Germany) from spheres were prepared and used. After fixation with 3.7% paraformaldehyde buffer and blocking with normal goat serum for 30 min, samples were incubated with a primary monoclonal antibody cocktail against certain antigens that were obtained from mouse or rabbit hosts for 1 hour at room temperature. After washing with PBS, slides were incubated for 1 hour with secondary fluorescent labeled antibody cocktail consisting of 488 nm goat anti-rabbit and 594 nm goat anti-mouse (life technologies, Carlsberg, CA, USA). The antibody list can be found in Supplementary Table S2. Cells were washed again and slides were coverslipped with the SlowFade® gold antifade mounting media with 4′,6-Diamidin-2-phenylindol (DAPI) (life technologies). Image analysis was performed with Olympus Basic BX51 microscope.

### Copy number profiling

Genome-wide somatic copy number aberrations (SCNA) were established using low-coverage whole genome sequencing. Shotgun libraries were prepared using the TruSeq DNA Nano LT Sample preparation Kit (Illumina, San Diego, CA, USA) with slight modifications to the manufacturer’s protocol. Detailed description can be found in the Supplementary materials file. Copy number analysis was performed as previously described^37^ and is further described in Supplementary materials file.

### Colony formation assay

Sorted cells were seeded at 200 cells per well in 6-well plates with DMEM/F12 media containing FBS and incubated for 8 days. Additional culture media was added every 3 days. Cells were fixed with ice cold methanol for 10 min, following the staining with 0.5% crystal violet (Sigma Aldrich, Vienna, Austria) solution for 10 minutes. Staining was rinsed off with ddH_2_O. Colonies were counted with ImageJ software.

### Soft agar assay

0.3% soft agar (Sigma Aldrich) in media was mixed with 800 sorted cells and plated onto a 0.6% solid agar layer in a 24-well plate^38^. After 3 weeks of sphere formation, the plate was stained with 0.005% crystal violet (Sigma Aldrich) and the total numbers of colonies were counted under a light microscope Olympus Basic BX51.

### Sphere formation assay

900 cells were sorted into a 24-well ultra-low attachment plate and cultured in sphere formation media for 3 weeks according to the protocol of Lombardo *et* *al*.^39^. Cell culture media was added only once a week. Formed spheres >50 µm were counted under the microscope. Sphere formation assays were performed in 6 technical replicates and three independent experiments (biological replicates) were performed.

### Ex ovo chorioallantoic membrane (CAM) assay

Fertilized white leghorn chicken eggs from local hatchery were incubated at 37.6 °C and 70% humidity (J. Hemel, Brutgeräte GmbH and Co. KG, Verl-Kaunitz, Germany). The egg shell was cracked on day 3 of chick development and the embryo decanted to a sterile dish. On day 10, 1*10^6^ cells (4:1 Matrigel matrix; BD, Biosciences) were grafted in the centre of a 5 mm silicon ring on the surface of the CAM^40^. Xenografts were allowed to develop for 3 days, photographed using a stereo-microscope (Olympus SZX16, Hamburg, Germany) and then excised with the surrounding CAM 4 day after seeding, fixed in 4% paraformaldehyde, paraffin embedded, cut in 4-μm sections. Every 10th section was stained with HE to find the area of interest. For the immunofluorescent analysis of the tumors, the same markers were used as for the primary culture cells. To distinguish the chicken cells, every section was co-stained with Lens Culinaris Agglutinin (LCA) antibody (Vector Laboratories, CA, USA)^41^.

### Drug treatment viability assay

On day one, 1000 cells were seeded into 96-well plates. On day two cells were treated with chemotherapeutics including Cisplatin (Accord Healthcare, Salzburg, Austria), Pemetrexed (Lilly, Indianapolis IN, USA) and Salinomycin (Sigma Aldrich) at varying concentrations. Untreated cells and cells treated with solvent of chemotherapeutics (vehicle control VC) served as controls. After 72 h, Prestoblue (Invitrogen, Vienna, Austria) was added according to manufacturer’s instructions and the fluorescence emission was measured on the FLUOstar omega plate reader (BMG-Labtech, Ortenberg, Germany). Half-maximal inhibitory concentrations (IC50) were calculated using GraphPad Prism6 non-linear regression analysis (variable Hill slope, four parameter fit). Experiments were done in five technical and three biological replicates.

### *In vivo* mouse experiments

These experiments were performed by an external facility (Epo, Berlin, Germany), which was completely blinded to the results of our study. All animal experiments were done in accordance with the United Kingdom Coordinating Committee on Cancer Research regulations for the Welfare of Animals and the German Animal Protection Law and with approval by the local responsible authorities (LaGeSo Berlin, G0030/15). Female NOD/SCID mice (Taconic, Cologne, Germany), kept under conditions summarized in Supplementary Table S4, were stratified at day 0 into two groups, each with 3 mice. For subcutaneous injection, 1 × 10^7^ cells were re-suspended in 100 µL PBS/Matrigel (1:1) and transplanted subcutaneously to the left flank of each mouse. LT22s cells were transplanted to mice of group A, while LT22a cells were transplanted to mice of group B. Tumor diameters were measured two times a week with a caliper. Tumor volumes were calculated according to V = (length × (width)^2^)/2. For calculation of the relative tumor volume (RTV), the tumor volumes at each measurement day were related to the day of first treatment. Mice were sacrificed by cervical dislocation and liver and lung tissue was removed for inspection of metastatic spread. After isolation of genomic DNA from two samples of each tissue, a human specific DNA was performed amplifying a fragment of the alpha satellite region of human chromosome 17. Indicated are the ΔCt values, which were calculated by subtracting the experimental mean Ct-value from that of the negative control (mouse tissue). Genomic DNA of the human mammary carcinoma xenograft 3366 was used as positive control, and water as negative control.

### Statistical analysis

Statistical significance was evaluated by either Student’s t-test or analysis of variance (ANOVA). To control for multiple testing, Sidak adjustment method was used. All p values were two-sided and considered statistically significant when <0.05. All statistical analysis was performed using GraphPad Prism 6 (GraphPad Software, La Jolla, CA, USA).

## Electronic supplementary material

Supplementary information
