# Supplementary material for: Primary patient-derived lung adenocarcinoma cell culture challenges the association of cancer stem cells with epithelial-to-mesenchymal transition
Source: Sci Rep. 2017 Aug 30;7:10040. doi: 10.1038/s41598-017-09929-0 (PMC5577216; doi:10.1038/s41598-017-09929-0)
Supplement: Supplementary file 1 — Supplementary information [file 41598_2017_9929_MOESM1_ESM.pdf]

# Primary patient-derived lung adenocarcinoma cell culture challenges the association of cancer stem cells with epithelial-to-mesenchymal transition.

Verena Tiran[1], Joerg Lindenmann[2], Luka Brcic[3], Ellen Heitzer[4], Stefanie Stanzer[1], Nassim Ghaffari Tabrizi-Wizsy[5], Elvira Stacher[3,6], Herbert Stoeger[1], Helmut H. Popper[3], Marija Balic[1,7#*] and Nadia Dandachi[1,8#*]

[1]Division of Oncology, Department of Internal Medicine, Medical University of Graz, A-8036 Graz, Austria

[2]Division of Thoracic and Hyperbaric Surgery, Medical University of Graz, A-8036 Graz Austria

[3]Institute of Pathology, Medical University of Graz, A-8036 Graz Austria

[4]Institute of Human Genetics, Medical University of Graz, A-8010 Graz, Austria

[5]SFL Chicken CAM Lab, Institute of Pathophysiology and Immunology, Medical University of Graz, A-8010 Graz, Austria

[6]Ludwig Boltzmann Institute for Lung Vascular Research, A-8010 Graz

[7]Research Unit Circulating Tumor Cells and Cancer Stem Cells, Medical University of Graz, A-8036 Graz, Austria.

[8]Research Unit Epigenetic and Genetic Cancer Biomarkers, Medical University of Graz, A-8036 Graz, Austria

# These authors shared senior authorship of this work

**Supplementary Figure S1.**

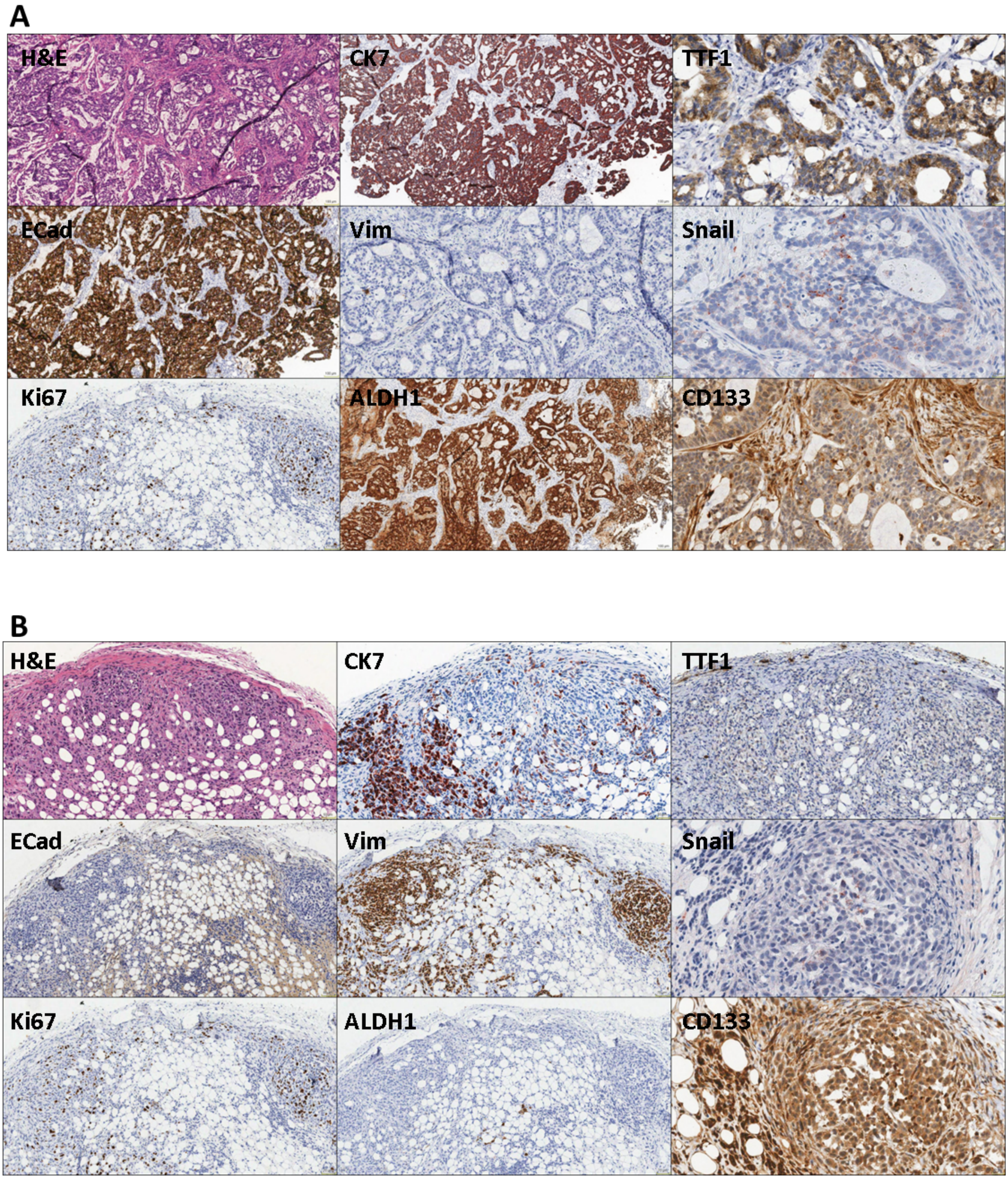


**Supplementary Figure S1.** Representative images of H&E and IHC staining of LT22s (**A**) and LT22a (**B**) xenografts. Scale bars between 20 and 300µm (insets).

## Supplementary methods

### *Tumor cell isolation in detail*

Tumor tissues were cut into small (3-5 mm) pieces and collected in gentleMACS C-tubes (Miltenyi Biotec) containing PBS and Collagenase B 2 mg/mL (Roche). C-tubes were then connected to the gentleMACS dissociator and tumor dissociation was run with the program for human tumor number 1. Minced tumors were incubated in the C-tubes using a MACSmix Tube Rotator (Milteny Biotec) under continuous rotation for one hour at 37°C. Following incubation, a second dissociation step was performed on the gentleMACS using the human tumor program number 2. The resulting single cell suspension was then filtered through a 70 µm cell strainer and centrifuged at 300g for 5 min. Red blood cells were lysed using ammonium chloride solution (Stemcell Technologies) and cells were then transferred to ultra-low attachment flasks (Corning, Acton, MA, USA) with CSC media.

### *Immunohistochemistry (IHC)*

After deparaffinization and rehydration, sections were pretreated with low pH retrieval solution (Dako, Vienna, Austria) for 10 min in the microwave. For the immunostaining the Ultravision LP Detection System based on horse radish peroxidase (HRP) Polymer and 3,3'-Diaminobenzidine (DAB) Plus Chromogen (Thermo Scientific, Waltham, MA USA) was used according to manufacturer's protocol. Briefly, after blocking with ultra V block for 5 min, sections were incubated with the antibody of interest. The signal was enhanced with the incubation of a primary antibody enhancer. Sections were first incubated with HRP polymer and then with DAB plus solution. The sections were counterstained with an attenuated HE staining and mounted with Aquatex (Merck, Darmstadt, Germany).

### ***Quantitative real time polymerase chain reaction (qRT-PCR)***

mRNA was isolated from $5*10^5$-$1*10^6$ cells with the RNeasy Mini Kit (Qiagen, Hiden, Germany) according to the manufacturer's protocol. The isolated RNA was eluted in 30 µL RNase-free water. Reverse transcription of 1 µg of RNA into cDNA was done with the QuantiTect reverse transcription kit (Qiagen) according to the manufacturer's instructions. mRNA expression was investigated by qRT-PCR with SYBR Green assay (Roche, Vienna, Austria) on a Light Cycler 480 (Roche) using 96 well plates. Reactions were performed in a total volume of 20 µL containing 1x Mastermix SYBR green I (Roche), 25 µM of each primer and 20 ng cDNA. A PCR program was run for 45 cycles starting with the denaturation step at 95°C for 10 sec, then 60°C for 20 sec, and an elongation step at 72°C for 15 sec. For quality control, it ended with a melting step of the product. A non-amplification control and a non-target control were included in each plate as technical controls. qRT-PCR reactions were performed in duplicates and cycle threshold values were averaged. Calculation of expression values was done using the qBase$^{plus}$ software (Biogazelle, Gent, Belgium)[1]. Glycerinaldehyd-3-phosphat-Dehydrogenase (GAPDH) and TATA-Box Binding Protein (TBP) were determined as appropriate reference genes using the geNorm module in qBase$^{plus}$ and were used to normalize gene expression levels. The gene expression levels in spheres were compared to expression in adherent cells. Primers are summarized in the **Supplementary Table S3**.

### ***Copy number profiling in detail***

Depending on the DNA concentrations, 50-100 ng of DNA from sorted cell fractions and 1-2 µg of DNA from tumor samples were fragmented in 130 µL using the Covaris System (Covaris, Woburn, MA, USA). After concentrating the volume to 50 µL end repair, A-tailing and adapter ligation were performed following the manufacturer's instructions. For selective amplification of the library fragments that have adapter

molecules on both ends, we used 8-15 PCR cycles. Libraries were quality checked on an Agilent Bioanalyzer using a DNA 7500 Chip (Agilent Technologies, Santa Clara, CA, USA) and quantified using qPCR with a commercially available PhiX library (Illumina) as a standard. Six libraries were pooled equimolarily and sequenced on an Illumina MiSeq in a 150bp single read run. On the completion of the run, data were base-called, demultiplexed on the instrument (provided as Illumina FASTQ 1.8 files, Phred+33 encoding), and FASTQ format files in Illumina 1.8 format were used for downstream analysis. Copy number analysis was performed as previously described [2]. Briefly, low-coverage whole-genome sequencing reads were mapped to the pseudo-autosomal-region (PAR)-masked genome and reads in different windows were counted and normalized by the total number of reads. The read counts were further normalized according to the GC-content using LOWESS-statistics. In order to avoid position effects, the sequencing data were normalized with GC-normalized read counts of a set of 30 non-malignant control samples [2]. Subsequently, segments of similar copy-number values were generated by applying circular binary segmentation and gain and loss analysis of DNA. For each segment, a z-score was calculated, that compared GC-corrected read counts for samples and controls [2].

***Authentication of cell populations***

In order to verify the same origin of different cell populations, passages and tumor samples, STR profiling and analysis of mitochondrial DNA (mtDNA) was performed. Mitochondrial genomic sequences were extracted from whole genome sequencing data obtain from the copy number profiling assay and haplotypes were compared for each sample. For STR analysis 0.7 ng of extracted DNA were amplified with the PowerPlex 16HS System (Promega, Mannheim, Germany) according to manufacturer´s instruction on a thermocycler MyCycler (Biorad, Vienna, Austria). In

this analysis 16 STR loci can be evaluated such as Penta E, D18S51, D21S11, TH01, D3S1358, FGA, TPOX, D8S1179, vWA, Amelogenin, Penta D, CSF1PO, D16S539, D7S820, D13S317 and D5S818. The amplified fragments were detected with a capillary electrophoresis on the 3730 Genetic Analyzer (Applied Biosystem, Vienna, Austria).

**Supplementary Table S1**: STR- analysis of LT22Leuko and LT22 primary tumor in FFPE compared to LT22s and LT22a DNA:

| STR Locus | LT22 Leukos | LT22 FFPE | LT22s | LT22a |
|---|---|---|---|---|
| **D3S1358** | 16, 17 | 16, 17 | 16 | 16 |
| **TH01** | 6, 7 | 6, 7 | 7 | 7 |
| **D21S11** | 32.2 | 32.2 | 32.2 | 32.2 |
| **D18S51** | 15, 17 | 15, 17 | 15, 17 | 15, 17 |
| **Penta E** | 13, 14 | 13, 14 | 13, 14 | 13, 14 |
| **D5S818** | 11 | 11 | 11 | 11 |
| **D13S317** | 12 | 12 | 12 | 12 |
| **D7S820** | 8 | 8 | 8 | 8 |
| **D16S539** | 12, 14 | 12, 14 | 12, 14 | 12, 13, 14 |
| **CSF1PO** | 12, 13 | 12, 13 | 13 | 13 |
| **Penta D** | 9, 11 | 9, 11 | 11 | 11 |
| **Amelogenin** | X, Y | X, Y | X | X |
| **vWA** | 16, 17 | 16, 17 | 16, 17 | 16, 17 |
| **D8S1179** | 11, 13 | 11, 13 | 13 | 13 |
| **TPOX** | 8, 9 | 8, 9 | 8 | 9 |
| **FGA** | 19, 22 | 19, 22 | 19, 22 | 19, 22 |

**Supplementary Table S2**: Antibodies used for IHC/IF and flow cytometry:

| AB/Target | Clone | Company | Cat. No. | Dilution | Reactivity | Host |
|---|---|---|---|---|---|---|
| | | | IHC /IF staining | | | |
| **ALDH1** | 44/ALDH | BD | 611195 | 1:100 | human | mouse |
| **aSMA** | IA4 | Sigma-Aldrich | A2547 | 1:800 | human | mouse |
| **CD133/1** | AC133 | Miltenyi Biotec | 130-090-422 | 1:50 | human | mouse |
| **CK7** | OV-TL 12/30 | Dako | M7018 | 1:500 | human | mouse |
| **Snail** | E-18 | Santa Cruz | sc-10432 | 1:100 | human | goat |
| **TTF1** | sp141 | Ventana | 790-4756 | Ready to use | human | rabbit |
| **EpCam** | Ber-EP4 | Dako | M0804 | 1:1000 | human | mouse |
| **Vimentin** | 3B4 | Dako | M7020 | 1:100 | human | mouse |
| **Ki67** | MIB-1 | Dako | M7240 | 1:100 | human | mouse |
| **panCK** | polyclonal | Dako | Z0622 | 1:300 | cow | rabbit |
| **E-Cadherin** | HECD-1 | Invitrogen | 13-1700 | 1:200 | human | mouse |
| **Alexa Fluor 488** | - | Life Technologies | A-11034 | 1:300 | rabbit | goat |
| **Alexa Fluor 594** | - | Life Technologies | A-11005 | 1:300 | mouse | goat |
| | | | Flow cytometry and cell sorting | | | |
| **ALDH1** | Enzyme reaction | Stemcell Techn. | 01700 | 5µL for $1*10^6$ cells | - | - |
| **CD133- APC** | AC133 | Miltenyi Biotec | 130-098-829 | 10µL for $10^6$ cells | human | mouse |
| **EpCam- PE** | HEA125 | Miltenyi Biotec | 130-098-115 | 3µL for $1*10^6$ cells | human | mouse |

**Supplementary Table S3:** Primer for qRT-PCR

| Genes | Forward primer (5´-3´) | Reverse primer (5´-3´) | Amplicon (bp) |
|---|---|---|---|
| | | HKG | |
| GAPDH [a] | CCACTCCTCCACCTTTGAC | ACCCTGTTGCTGTAGCC | 102 |
| TBP [a] | CGGTTTGCTGCGGTAATC | TCTGGACTGTTCTTCACTCTTG | 108 |
| | | Cancer stem cell markers | |
| ALDH1 [a] | AGAAGGAGATAAGGAGGAT | AATCAGCCAACTTGTATAATAG | 125 |
| CD133 (PROM 1) [b] | AGAGCTTGCACCAACAAAGTACAC | AAGCACAGAGGGTCATTGAGAGA | 91 |
| | | Stem cell markers | |
| OCT4 [c] | GACAACAATGAAAATCTTCAGGAG | CTGGCGCCGGTTACAGAACCA | 216 |
| SOX2 [c] | GCACATGAACGGCTGGAGCAACG | TGCTGCGAGTAGGACATGCTGTAGG | 207 |
| NANOG [c] | CAGCTGTGTGTACTCAATGATAGATTT | ACACCATTGCTATTCTTCGGCCAGTTG | 179 |
| | | EMT markers | |
| E-Cadherin (CDH1) [b] | TGAGTGTCCCCCGGTATCTTC | CAGTATCAGCCGCTTTCAGATTTT | 87 |
| Vimentin [c] | CAACCTGGCCGAGGACAT | ACGCATTGTCAACATCCTGTCT | 113 |
| Fibronectin [c] | CCGCCGAATGTAGGACAAGA | TGCCAACAGGATGACATGAAA | 100 |
| N-Cadherin [c] | GACGGTTCGCCATCCAGAC | TCGATTGGTTTGACCACGG | 67 |
| | | EMT transcription factors | |
| SNAIL [c] | GCTGCAGGACTCTAATCCAGAGTT | GACAGAGTCCCAGATGAGCATTG | 130 |
| SLUG [c] | GCGATGCCCAGTCTAGAAAA | GCAGTGAGGGCAAGAAAAAG | 203 |
| TWIST [c] | GGAGTCCGCAGTCTTACGAG | TCTGGAGGACCTGGTAGAGG | 201 |
| Zeb1 [c] | GCCAATAAGCAAACGATTCTG | TTTGGCTGGATCACTTTCAAG | 101 |
| Zeb2 [c] | CCCTTCTGCGACATAAATACG | TGTGATTCATGTGCTGCGAGT | 192 |

bp: size of amplicon in basepairs

[a] http://www.ncbi.nlm.nih.gov/tools/primer-blast/

[b] http://www.rtprimerdb.org/

[c] Palafox M. et al. Cancer Research 2012

**Supplementary Table S4:** Conditions for mouse experiments

| Subject | Conditions |
|---|---|
| **Animal strain** | Female NOD/SCID |
| **Age** | 9-12 weeks |
| **Body weight** | 21.4 to 24.1 g (mean 22.5 ± 1.1 g) at time of cell inoculation |
| **Supplier** | Taconic, Cologne, Germany |
| **Environmental conditions** | Strictly controlled and standardized barrier conditions, IVC System Tecniplast DCC (TECNIPLAST DEUTSCHLAND GMBH) |
| **Caging** | Macrolon Type-II wire-mesh bottom |
| **Feed Type** | Ssniff NM, Soest, Germany |
| **Drinking water** | Autoclaved tap water in water bottles (acidified to pH 4 with HCl) |
| **Feeding and drinking time** | Ad libitum 24 hours per day |
| **Light period** | Artificial; 12-hours dark/12 hours light rhythm (light 06.00 to 18.00 hours) |
| **Health control** | The health of the mice was examined at the start of the experiment and twice per day during the experiment |
| **Identification** | Ear mark and cage labels |

## Supplementary references


1 Hellemans, J., Mortier, G., Paepe, A. D., Speleman, F. & Vandesompele, J. qBase relative quantification framework and software for management and automated analysis of real-time quantitative PCR data. . *Genome Biol.* **8** (2007).

2 Heitzer, E. *et al.* Tumor-associated copy number changes in the circulation of patients with prostate cancer identified through whole-genome sequencing. *Genome Med.* **5** (2013).
